# Progression in Ph-Chromosome-Negative Myeloproliferative Neoplasms: An Overview on Pathologic Issues and Molecular Determinants

**DOI:** 10.3390/cancers13215531

**Published:** 2021-11-04

**Authors:** Elena Sabattini, Marco Pizzi, Claudio Agostinelli, Clara Bertuzzi, Carlo Alberto Sagramoso Sacchetti, Francesca Palandri, Umberto Gianelli

**Affiliations:** 1 Haematopathology Unit, IRCCS Azienda Ospedaliero-Universitaria di Bologna, 40138 Bologna, Italy; claudio.agostinelli@unibo.it (C.A.); clara.bertuzzi@aosp.bo.it (C.B.); carloalberto.sagramososacchetti@aosp.bo.it (C.A.S.S.); 2 Surgical Pathology and Cytopathology Unit, Department of Medicine—DIMED, University of Padua, 35121 Padua, Italy; marco.pizzi@unipd.it; 3 Department of Experimental, Diagnostic and Specialty Medicine, University of Bologna, 40126 Bologna, Italy; 4 Istituto di Ematologia “Seragnoli” IRCCS Azienda Ospedaliero-Universitaria di Bologna, 40138 Bologna, Italy; francesca.palandri@aosp.bo.it; 5 Pathology Unit, Department of Pathophysiology and Transplantation, University of Milan and IRCCS Fondazione Ca’ Granda, Ospedale Maggiore Policlinico, 20122 Milan, Italy; umberto.gianelli@unimi.it

**Keywords:** myeloproliferative neoplasms, WHO classification, progression, fibrosis, leukemia

## Abstract

**Simple Summary:**

The present review is meant to provide an updated overview on the progressions in Ph-chromosome negative MPN, with major focus on the histopathological changes identifiable in routine diagnostic practice on bone marrow biopsies. It integrates these issues with clinical parameters that define the risk of progression and the molecular determinants that are potentially involved in the transformation. The fibrotic and accelerated/leukemic types of progression are defined by the Who Classification, but laboratory changes may occur during the course of the disease, such as monocytosis or leukocytosis. These can impact on morphology and challenge the histologic diagnosis with potential risk of reclassification. Molecular investigations are becoming relevant for the management of these patients and profoundly changing and challenging our diagnostic approach, but histology remains a turning point for the diagnosis and classification of Ph-negative MPN and should remain the reference also in the event of unusual or discordant molecular findings.

**Abstract:**

Progression in Ph-chromosome-negative myeloproliferative neoplasms (MPN) develops with variable incidence and time sequence in essential thrombocythemia, polycythemia vera, and primary myelofibrosis. These diseases show different clinic-pathologic features and outcomes despite sharing deregulated JAK/STAT signaling due to mutations in either the Janus kinase 2 or myeloproliferative leukemia or *CALR*eticulin genes, which are the primary drivers of the diseases, as well as defined diagnostic criteria and biomarkers in most cases. Progression is defined by the development or worsening of marrow fibrosis or the progressive increase in the marrow blast percentage. Progression is often related to additional genetic aberrations, although some can already be detected during the chronic phase. Detailed scoring systems for clinical usage that are mostly applied in patients with primary myelofibrosis have been defined, and the most recent ones include cytogenetic and molecular parameters with prognostic significance. Additional different clinic-pathologic changes have been reported that may occur during the course of the disease and that are, at present, classified as WHO-defined types of progression, although they likely represent such an event. The present review is meant to provide an updated overview on progression in Ph-chromosome-negative MPN, with a major focus on the pathologic side.

## 1. Introduction

Myeloproliferative neoplasms (MPNs) are clonal hematopoietic stem cell myeloid neoplasms that are characterized by the proliferation and preserved differentiation of myeloid cell lineages. Excluding *BCR-ABL1*-positive chronic myeloid leukemia (CML), essential thrombocythemia (ET), polycythemia vera (PV), and primary myelofibrosis (PMF) are the most frequent MPNs [1].

All of these diseases more commonly develop in adults, with a favourable median survival of 20 years in ET and a median life expectancy of 14 and 6 years in PV and PMF, respectively [1,2].

Although our understanding of the biology of MPNs has increased greatly in recent years, Ph-neg MPNs still represent an enigma in terms of patho-physiology. Despite these diseases showing different clinic-pathologic features, outcomes, and risk of progression, they share a common hallmark of constitutively activated *JAK/STAT* signaling. This is primarily related to somatic point mutations in the *JAK2* gene on exon 14 (mostly occurring at codon 617 in the pseudokinase domain of the gene, with valine being substituted with phenylalanine aminoacid) or other activating *JAK2* mutations in exon 12 or more rarely to mutations in the myeloproliferative leukemia (*MPL)* gene or in exon 9 of the *CALR*eticulin (*CALR*) gene (of which 2 subtypes—type 1 and type 2—have been recognized) [1,3,4]. These genetic alterations are the primary drivers of these diseases, represent the objectives of the WHO-defined diagnostic criteria [1], and are helpful positive biomarkers in about 98% of patients with PV and in 85% to 90% of patients with ET and PMF, thus representing a major landmark of MPN diagnosis. They are mutually exclusive, and each MPN subcategory harbours either one, though at different incidences [1]; nonetheless, published data indicate that the type of driver genetic lesion may somehow imprint the clinical course, which is also within the same disease entity [5,6], and may differently influence prognosis, especially in PMF patients [7]. The presence of the *JAK2* mutation has supported the clinical usage of *JAK2* inhibitors, although so far, this has not shown the capability to ensure long-term remissions and/or modify the course of the disease: these observations likely indicate that other mechanisms concur with *JAK-STAT* signaling dysregulation in the development of and, above all, in the progression of MPNs. In fact, mutations have been identified in genes that have been considered as non-drivers for these diseases, which have been shown to be involved in other myeloid neoplasms, including those related to DNA methylation (*IDH1/2*, *TET2*, *DNMT3A*), chromatin remodeling (*ASXL1*, *EZH2*), RNA splicing (*U2AF1*, *SF3B1*, *SRSF2*), and DNA repair (*TP53*, *PPMID*) [8]. Such mutations are present in more than 80% PMF patients and have an impact on overall survival (OS) and leukemia-free-survival and are associated with a higher risk of fibrotic progression [8,9] that is also independent of the prognostic score that is applied (see below). In particular, the *ASXL1*, *SRSF2*, *EZH2*, and *IDH1/IDH2* mutations are considered high–molecular risk (HMR) mutations, and their prognostic relevance is also dependent on their number in an individual patient [8,9,10,11]. Consistently, testing for mutations in *ASXL1*, *EZH2*, *TET2*, *IDH1/2*, *SFSF2,* and *SF3B1* genes is recommended for prognosis according to the WHO classification [1], but testing also serves diagnostic purposes in cases of triple-negative MPNs. Notwithstanding, it should be underlined that other unrelated conditions such as age, epigenetic events, clonal hematopoiesis, and environmental and host factors intervene in MPN phenotype and prognosis [12,13,14]. Ph-neg MPNs have historically been regarded as chronic diseases that share the tendency of undergoing a myelofibrotic and/or leukemic transformation, although the course of the disease is highly variable regardless the therapy type, with some patients experiencing a slowly progressing disease and others showing a faster transformation to overt myelofibrosis (MF) or acute leukemia. The WHO classification [1] recognizes two basic types of progression in MPNs: fibrotic and accelerated/leukemic. The former requires a grade 2 or 3 fibrosis (out of 3 defined grades), as defined by Thiele et al. [15], and the latter requires an increase in the percentage of blasts in the bone marrow or blood and is subdivided into an accelerated phase (AP) and a blast phase (BP), which are defined by 10–19% and ≥20% blasts, respectively.

Whatever the type, the concept of progression relates to the definition of its risk of occurrence, which are defined by clinical, molecular (genetic and cytogenetic), and pathologic parameters: in this review, we will provide a brief overview of the available scoring systems for risk definition and will place major focus on the pathologic features and main risk-factors and prognostic significance of myelofibrotic and leukemic progressions as well as of other rare types of possible MPN evolution.

The following paragraphs will provide a definition for clinical risk progression and will give a brief overview of the scoring systems that have been developed thus far:

Many scoring systems have been proposed in recent years, with the most updated versions integrating recent molecular data that have greatly enhanced our biologic understanding of these diseases. Most of them apply to PMF patients. The considered parameters include laboratory and blood work up data, presence and/or type of gene driver and non-driver mutations, and karyotype. The presence of the type-1/type-1 like *CALR* mutation in PMF relates to a significantly superior overall survival (OS) compared to the *JAK2* (HR, 2.6, 95% CI 1.9–3.5), type-2/type-2 like *CALR* (HR 2.5, 95% CI 1.4–4.4), or *MPL* (HR 1.8, 95% CI 1.1–2.9) mutations and triple-negative mutational status (HR 2.4, 95% CI 1.6–3.6), with the latter four conditions having a similar impact on outcome [16,17,18]. The *ASXL1*, *SRSF2*, *EZH2*, and *IDH1*/*IDH2* mutations are considered a high-molecular risk (HMR) category [8,9,10,11].

The International Prognostic Scoring System (IPSS) was published in 2009 [19], and it is based on clinical parameters at diagnosis, including age (over 65 years; score: 1 point), hemoglobin (<10 g/dL; score: 2 points), WBC count (>25 × 10^9^/L; score: 1 point), peripheral blood blasts (≥1%; score: 1 point), and constitutional symptoms (score 1 point), while the Dynamic IPSS (DIPSS) [20], which was proposed one year, later defines risk factors both at diagnosis and during the disease course. This system allows the recognition of four risk classes (class 0: low risk; 0 factors; OS 11.3 years, class 1: intermediate-1 risk; 1 factor; OS 7.9 years, class 2: intermediate-2 risk; 2 factors; OS 4 years, class 4: high risk; ≥3 factors; OS 2.3 years) with a progressive increase in the risk of progression to leukemia. The IPSS/DIPSS were further refined as DIPSS-plus [21], which included the addition of the karyotype status (with favourable and unfavourable subgroups, the latter including complex karyotype or only one or two abnormalities, including +8, 7/7q-, i(17q), 5/5q-, 12p-, inv(3), or 11q23 rearrangement), red cell transfusion need, platelet count (lower than 100 × 10^9^/L), and circulating blast percentage (≥1%). In 2018, the Mutation-Enhanced International Prognostic Scoring System 70 (MIPSS70) and its karyotype-implemented version MIPSS70+ [22] were published. Once again, the were specifically devoted to PMF patients aged ≤70 years of age, thus making these patients eligible for transplantation. While both considered the high-risk mutations in the *ASXL1*, *SRSF2*, *EZH2*, *IDH1*, and *IDH2* genes and the presence of the *CALR* type 1/like mutation (as favourable factor), the MIPSS70+ also included a two-tiered cytogenetic risk parameter (unfavourable vs. favourable) and a grade of bone marrow fibrosis while reducing the clinical risk factors of the MIPSS70 from 5 to 3 (hemoglobin <10 g/dL, circulating blasts ≥2%, constitutional symptoms). A further refined version of the MIPSS70+ was proposed shortly thereafter and was termed MIPSS70-plus version 2.0 [23], which more specifically stratified into five-tiered prognostic groups (very high risk: ≥9 points, high risk: 5–8 points, intermediate risk: 3–4 points, low risk: 1–2 points, very low risk: 0 points). A Genetically Inspired Prognostic Scoring System (GIPSS) for PMF was recently proposed and included *CALR* type 1/type 1-like mutations as a favourable marker on the one hand and *ASXL1*, *SRSF2*, and *U2AF1* p.Q157 mutations as poor predictors on the other [24]. This approach was validated and was reported to surpass clinically based scoring systems [25]. Although the above-mentioned systems are applicable to PMF patients, risk predictors have also been defined for ET and PV [8,9,26,27,28].

As evidenced in the most recent prognostic scoring systems, next generation sequencing analyses (NGS) have progressively been introduced in daily practice, and their application is changing the diagnostic approach to MPNS profoundly. Three recently published papers [29,30,31] focused on this issue. Hasserjian and collaborators [29] summarized cases of non acute myeloid neoplasms submitted to the 2017 workshop of the Society for Hematopathology/European Association for Haematopathology: they underlined that both supportive and contradictory results can be obtained with NGS and that the latter may sometimes challenge a WHO-defined classification of the disease. The conclusion was that the WHO-defined morphologic criteria [1] for myeloid neoplasms (and MPN in particular) also remain the point of reference in the event of unusual or discordant NGS findings. Notwithstanding, sequencing is strongly suggested, particularly in PMF patients, since it allows the further refinement of the risk-based stratification of patients, particularly those who are potentially eligible for transplantation. In ET and PV patients, NGS is basically applied at an investigational level and is not yet recommended by guidelines, although it will likely be a future direction in order to identify aberrancies that are related to higher risk or rapid evolution.

## 2. Myelofibrotic Progression

This event develops during the course of ET, PV, and early PMF that, respectively, evolve into post-ET MF, post-PV MF, and overt PMF. Fibrotic progression [1,32] rarely occurs in ET within 10 years (0.8–4.9%) but raises to a likelihood of 4–11% at 15 years, which is higher in PV on average, which both show progression at 10 years and after 15 years (4.9–6% and 6–14%, respectively). Being part of the natural history of PMF, progression from early/pre-fibrotic stage to overt PMF reaches the highest incidence of 50% at 5 years.

### 2.1. Pathologic Features

Progression towards a fibrotic stage is the most common situation seen in cases of these diseases, and progression is defined by major and minor diagnostic criteria (Table 1). The 0–3 scale grading system [15] is the most widely applied system and has good reproducibility among pathologists [33]. Grade 2 is defined [1] as a diffuse and dense increase in reticulin fibers with extensive intersections, occasionally with focal bundles of thick fibers that are mostly consistent with collagen and/or associated with focal osteosclerosis, while grade 3 is defined as a diffuse and dense increase in reticulin fibers with extensive intersections and coarse bundles of thick fibers that are consistent with collagen and are usually associated with osteosclerosis. The reference staining method that is used for detecting fibrosis is silver impregnation, but trichrome staining, which highlights collagen deposition better, can be also performed, particularly in patients who are enrolled in clinical trials or if silver impregnation staining does not easily allow the distinction between collagen and reticulin fibers, which may happen with some automated stainers. The WHO classification [1] also provides a semiquantitative 0–3 scale grading system for collagen deposition. The fibrotic process is associated with a profound remodeling of the bone trabeculae, which is also subject to grading by a semiquantitative grading system provided by the WHO classification [1]. Interestingly, we recently documented the prognostic significance of the complete evaluation of stromal changes in PMF according to the WHO proposal [34].

Although fibrosis is, by definition, a dynamic process that develops progressively, its degree is commonly fairly homogeneous throughout the bone marrow biopsy, particularly if the biopsy is performed in clinically advanced stages when the process is likely fully blown. Nonetheless, it is worth a reminder that bone crushing commonly occurs during sampling procedures, with an artefactual increase in the reticulin fiber density: such areas should not be considered when grading fibrosis, which should only be defined in cytologically preserved hematopoietic areas. During treatment with *JAK2* inhibitors a decrease in the degree of fibrosis has been reported in some cases, leading to a more uneven distribution of the fibers throughout the single bone marrow biopsy. This event can challenge a reliable and reproducible assessment of the final grading score. Kvasnicka et al. [35] proposed that the final score that should be assessed is the highest grade representing at least 30% of the marrow area.

**Figure 1:**
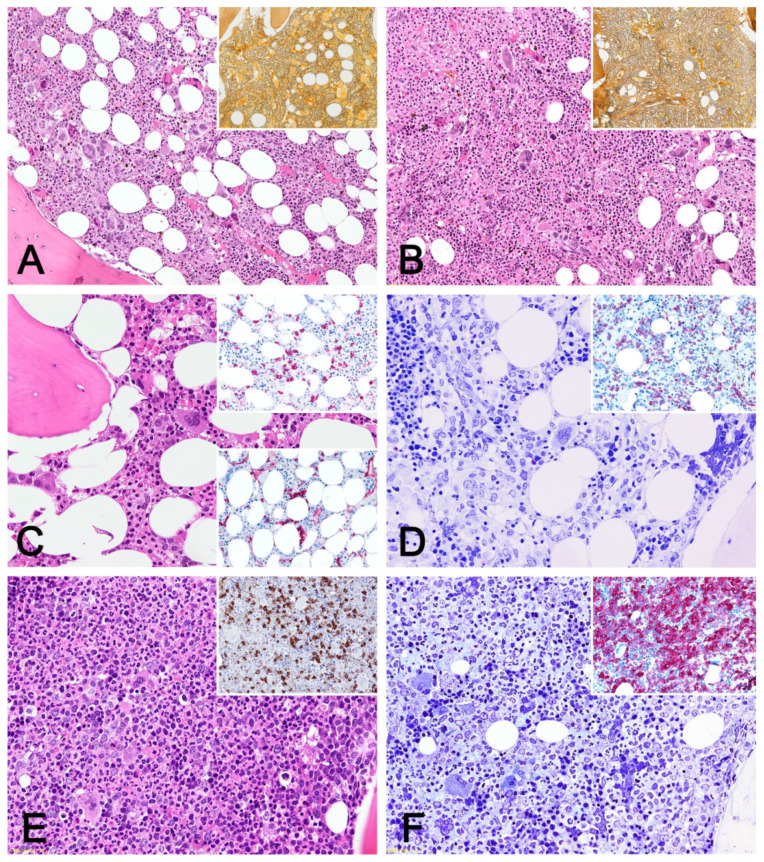
(**A**) Post-ET MF: hypercellular marrow with prominent granulopoiesis and expanded megakaryocytes; some of the latter look more ET-like than PMF-like; H&E ×10; inset: grade 2 fibrosis; Gomori silver impregnation ×10. (**B**) Post-PV-MF: hypercellular marrow with prominent granulopoiesis and expanded megakaryocytes; most of the latter show MF-like morphology although variability occurs; H&E ×10; inset: grade 3 fibrosis; Gomori silver impregnation ×10. (**C**) ET in accelerated phase: histology shows features of typical ET with mild increase in precursors; H&E, ×10; inset: the CD34 staining highlights increases in positive blastic cells that overall account for more than 10% cellularity (12% on the whole biopsy) and also shows occasional clustering features; ×10. (**D**) PMF *JAK2*-mutated case in blastic phase: myeloid maturation arrest and increase in blasts; residual erythroid precursors and few atypical megakaryocytes can be observed; H&E ×10; inset: CD34 positive blasts accounting for 35–40% cellularity; ×10. (**E**) *JAK2* positive PMF case with subsequent development of monocytosis: hypercellular marrow with increase in granulopoietic precursors and monocytes with CMML-like features; H&E ×10; inset: increase in CD14 positive monocytes; ×10. (**F**) PV with late development of leukocytosis: expansion of granulopoietic cells and erythroid precursors; Giemsa ×10; inset: excess of myeloperoxidase positive granulopoetic cells with preserved maturation; ×10.

The morphologic pictures of bone marrow in advanced myelofibrosis do not significantly differ between post-ET or post-PV MF (Figure 1A,B) and overt PMF cases, and this justifies why the WHO classification [1] requires knowledge of the patients’ previous history of a chronic-phase MPN to assess fibrotic progression. At the stage of advanced fibrosis, the cellularity is more often normal or moderately to highly decreased, but it is often unevenly distributed, demonstrating alternating hypercellular and depleted areas. Myeloid cells mostly maintain a high myeloid/erythroid ratio, and the reduced erythropoiesis is often only represented by early precursors. Megakaryocytes occur in clusters and show marked atypia and maturation defects. Along with the severe fibrosis, vascularity is increased with abundant dilated vessels, within which clusters of hematopoietic cells can be seen and where the bone trabeculae look thickened and abnormally shaped. Nonetheless, in a comparative study conducted by Boiocchi et al. [36], a trend for megakaryocytes to retain the original cytology was observed: in most post-PV MF biopsies, at least some megakaryocytes still maintained polymorphic features (Figure 1A,B) and demonstrated a lesser degree of clustering compared to overt PMF samples, where most megakaryocytes showed severe maturation asincrony and poorly lobulated nuclei.

### 2.2. Predictors of Progression

Risk factors for myelofibrotic progression have been reported and include older age; longer disease period; greater disease burden (as defined by leucocytosis, thrombocytopenia, anemia, palpable splenomegaly); higher *JAK2* allele burden (for PV); detection of *SRSF2*, *U2AF1*, and *ASXL1* mutations; and cytogenetic abnormalities (12p abnormality, acquired loss of heterozygosity of chromosome 1p) [19,20,21,37,38,39,40,41,42,43]. The median span of time to progression was reported to be approximately 11 years, which is longer than the time to progression for *CALR*-mutated than for *JAK2*-mutated ET, PV, and triple-negative ET (for which median times are 12.1, 8.4, 11.0, and 8.2 years, respectively) [43,44].

### 2.3. Grades of Fibrosis and Prognostic Significance

Something that is somewhat surprising is that a severe (≥2 of 0–3 scale system) grade of fibrosis has only recently been included as a parameter in the prognostic scoring systems for PMF and has only been specifically introduced into the MIPSS70+ [23]. Truly, in 2008, Vener C et al. [45] had already documented the prognostic significance of the evaluation of the grade of marrow fibrosis—defined according to the European Consensus [15]—in comparison to previous prognostic scoring systems. More recently, Gianelli et al. [46,47] also matched the grade of fibrosis [15] and the IPSS [19] in 196 bone marrow biopsies from PMF patients: not unexpectedly, not only did higher grades of fibrosis result in shorter survival probability, but the grade of fibrosis allowed the further stratification of the considered patients into different survival probability subsets within each IPSS risk category. Particularly, the higher the IPSS category, the stronger the power of the fibrotic grade to define prognostic subsets. In 2016, in a larger case series of 490 patients, Guglielmelli et al. [48] confirmed that the combination of the fibrosis grade with the IPSS scores could more accurately predict survival. This was also documented in an age-independent model that excluded older age, which is a strong inferior survival predictor in PMF. What is noteworthy is that these observations were of significant clinical relevance since they mostly impacted the lower risk categories, which include patients who do not benefit of the established treatment guidelines. Shortly after these studies were published, these finding were affirmed in a wide collaborative work [49] that confirmed that patients with overt PMF were enriched in cases with unfavourable clinical features (anemia, thrombocytopenia, leukopenia, higher blast count, symptoms, large splenomegaly), unfavourable karyotype, and high-risk mutations. In addition, these patients had a significantly shortened median survival compared to PMF patients at the pre-fibrotic stage (7.2 vs. 17.6 years).

As far as risk definition in post-ET and post-PV MF is concerned, all scoring systems adopted for PMF have shown limited applicability and reliability in stratifying these patients into risk groups [50,51]. In particular, Tefferi et al. [51] recently underlined that the main scoring systems that are in use could not segregate patients with intermediate-1 and low risks as well as those with intermediate-1 and intermediate-2 risks. In this scope, Passamonti et al. [52] developed a model that is applicable in patients with post-ET and post-PV MF, termed the Myelofibrosis SECondary Prognostic Model (MYSEC-PM), which is intended to vicariate IPSS and/or DIPSS for this subset of patients. It provides a final score based on the scores obtained from six independent predictors of inferior OS (hemoglobin <11 g/dL, circulating blast percentage ≥3%, no *CALR* mutation: 2 points; platelet count <150 × 10^9^/L, constitutional symptoms: 1 point; every year of age: 0.15 point) and defines four risk classes with progressive inferior OS. Different groups validated the predictive power of the MYSEC-PM in different case series [53,54,55], these case series also included patients treated with *JAK2* inhibitors [55].

Despite the assessment of a fibrotic progression implying a ≥2 grade of fibrosis, the precise definition and reporting of the reticulin fiber grade is mandatory in all types and stages of MPNs. In fact, grade 1 fibrosis presenting in ET and PV has been associated with an increased risk of MF transformation [8,9,40,48,49,56].

In the WHO classification [1], the concept of fibrotic progression is mostly addressed to ET and PV evolving into post-ET and post-PV MF. It could, however, also include PMF progression from the early/prefibrotic stage into overt PMF, although pathologists do not routinely report the late stage/overt PMF as “transformed or progressed” PMF. Although the presenting clinical and morphologic features of post ET/post-PV MF and overt-PMF are fairly similar, recent observations indicate differences between these two conditions [56,57,58], despite the former category being poorly defined in terms of prognostic risk assessment for the scarcity of genetic data. Boiocchi et al. [36] studied cytogenetics in some of their cases and observed that the post-PV MF group harboured a higher number of cases with complex karyotypes and showed higher degrees of karyotypic aberrations than the overt-PMF patient group. This was also confirmed by Masarova et al. [59] and Mora et al. [60], and Rumi et al. [61] observed a relationship between 9p chromosome alterations (mostly gains or uniparental disomy) and progression from PV to post-PV MF. Conversely, the occurrence of subclonal high-risk mutations seem to have similar rates in post-ET/post-PV MF patients and in PMF patients [62], although in the former, they do not appear to have a significant impact. Likewise, no major differences were reported regarding the predictive value of the driver mutations between post-ET and post-PV MF and PMF [62].

## 3. Leukemic Progression

The development of secondary acute leukemia (AL) is another recognized type of MPN progression. Two phases can be distinguished: an accelerated phase (AP) and a blastic phase (BP). The WHO classification [1] defines the former as an MPN with 10–19% blasts (Figure 1C) and the latter as an MPN with ≥20% blasts in the marrow or peripheral blood (Figure 1D). Evolution from AP to BP is observed. The evolution to BP is frequently unpredictable and is an almost invariably fatal event. Allogeneic hematopoietic stem cell transplantation is the only therapy that provides prolonged survival and a potential cure, but few patients can benefit from this option. Considering AP and BP collectively [1,32,63,64], ET once against the lowest risk of evolution, with 0.7–3% of ET patients developing AP/BP at 10 years and with 2.1–5.3% developing AP/BP at 15 years. Percentages comprised between 2.3 and 14.4, and 5.5 and 18.7 of PV patients evolve at 10 and 15 years, respectively. PMF shows leukemia progression in 10–20% of cases within 10 years.

### 3.1. Pathologic Features

The histologic pictures of acute progression in MPN can be variable. Blasts can either represent whole-marrow cellularity or may only represent partial infiltration. Fibrosis is also not necessarily present because ET and PV can develop BP also without passing through a fibrotic phase. Blasts are more commonly of myeloid origin and are CD34-positive, with possible heterogeneous distribution, clustering, and/or peri-endosteal location. The pathologic diagnostic approach and phenotypic algorithm applied both during flow cytometry and during immunoistochemistry do not differ from the standard ones for de novo AML, although they do not significantly impact patient management. In cases with significant fibrosis, the immunohistochemical quantification of CD34-positive blasts is mandatory, especially if BM aspirate smears are dry tap or hemodiluted. Megakaryoblastic differentiation in MPN-BP represents up to 25% of cases: its recognition can be challenging since megakaryoblasts are mostly negative for CD34 and are often hardly distinguishable from dysplastic megakaryocytes. The latter are common in progressing marrows, especially if they are also fibrotic. In such instances, in order to avoid the overdiagnosis of leukemic transformation, the presence of megakaryoblast sheets is required for a diagnosis of megakaryoblastic BP since they are absent in the chronic and accelerated phases [65]. Purely erythroid differentiation is rare, as is extramedullary location, which is as myeloid sarcoma, which can also represent the onset disease presentation [66,67]. Nucleophosmin-mutated AML has been reported as being an exceptional BP of a Ph-negative MPN, particularly in patients with PMF [68,69]. The residual marrow, if present, commonly shows features of delayed maturation with an excess of immature precursors over terminally differentiated cells (Figure 1D); features of dysplastic changes have been observed in up to 88% bone marrow biopsies from PV patients with BP [70]. However, the megakaryocytes can preserve the myeloproliferative features of the previous chronic phase. Regardless of the presence of dysplastic features, the WHO classification [1] indicates that the terminology used for progressing CML should also be applied to Ph-negative MPN: therefore, the BP progression in Ph-negative MPN must be referred to as blast-phase MPN, without further specification.

### 3.2. Predictors of Leukaemic Transformation

The molecular mechanisms underlying progression are still largely unclear or unknown, which is also because of the usually long timeline that is necessary for genetic progression and the final emergence of one or more predominant clones commonly characterized by complex genetics [12,63].

The incidence of AL is increased in so-called triple-negative MPNs, which show a trend to later blastic progression [2]. Among patients with driver gene mutations, those that are *JAK2*-mutated show a higher potential towards BP [71,72,73,74]; however, the impact of a lower *JAK2* mutation allele burden on this type of progression was only reported in PMF patients [73]. Interestingly, however, the *JAK2* V617F mutation is frequently lost with transformation to BP, highlighting that the evolution might derive from either early subclones (even preceding the *JAK2* V617F mutation) or from independent *JAK2* V617F-negative clones [75]. On the other hand, the *JAK2* V617F mutation may—though rarely—occur also in de novo acute myeloid leukemia (AML). In a study by Aynardi et al. [76] that compared de novo and BP *JAK2* V617F-mutated AML, differences were recorded. In particular, the latter group had higher probabilities of having splenomegaly, MPN-like megakaryocytes in the residual marrow, a higher mean *JAK2* V617F-variant allele frequency at diagnosis, a complex karyotype, and the morphologic resurgence of MPN after from induction chemotherapy. Moreover, the concurrent presence of *JAK2*V617F and *JAK2* “variants” (non-V617F/non-exon 12 *JAK2* mutations) has recently been associated with a higher risk of leukemic transformation in PMF [77]. Interestingly, half of these patients also harboured *TP53* mutations. Conversely, a positive prognostic effect of *CALR* mutations has been reported, particularly of type-1 *CALR*, provided that it is not associated with *ASXL1* aberrancy, although the real prognostic impact of *CALR* subvariants is still controversial in this clinical setting [2,78,79,80]. More generally, the overall number of mutations frequently increase upon progression and have an impact on outcome [12]; however, at least some of the mutated clones that effectively drive the transformation may already be present in the chronic phase [81]. The mutational profiles of MPN-AP and MPN-BP are similar but are markedly different from those of both de novo AML and MPN-CP [71,82].

Most investigations relate to either the chronic or blastic phase of diseases, whereas data for MPN-AP are limited: this is unfortunate since the opportunity to define risk at this earlier stage could provide better patient management [83] because the rate of leukemic progression during the chronic phase MPNs has been reported to be much lower than that in MPN-AP (1% at 3 years and 3% at 10 years, *p* < 0.001).

The dismal prognosis of this condition is worse than that of de novo AML, and the risk factors and molecular events associated with this evolution can mostly be assessed by the available scoring systems. In general, advanced age, severe anemia, leukocytosis, circulating blasts >2%, thrombocytopenia, advanced bone marrow fibrosis, cytogenetic abnormalities, and the acquisition of ≥2 HRM are recognized as risk factors for leukemic transformation in many studies. Monocytosis has also been reported as a strong indicator of AP and inferior survival in patients with PMF [84,85]: in a large case series, the presence of an absolute monocytosis (≥1 × 10^9^/L and >3 × 10^9^/L) increased the hazard risk of leukemic transformation by 2- and 4-fold, respectively [85]. Dobrowolski and colleagues reported persistent basophilia in advanced-stage PMF patients as a risk for leukaemic transformation [86].

Unfavourable outcomes and a higher cumulative risk of BP occurrence in PMF patients with low risk were related to the presence vs. the absence of HRM (*ASXL1*, *IDH1*/2, *EZH2*, *SRSF2*) [87,88,89]. Nonetheless, the role of *ASXL1* mutation as the sole mutation has recently been debated, with the suggestion that its poor prognostic role may only be effective if associated with mutations in *TP53* or in other high-risk genes [90,91]. Additionally, something noteworthy is the observation by Bartels et al. [90], where among those MPN that developed a BP, *IDH1*/*IDH2* hot-spot mutations mostly co-occurred with *SRSF2* and *U2AF1* mutations. Given that *JAK2*/IDH-mutated mice do not develop overt leukemia [91], it is possible that *IDH1*/*IDH2* are not key-genes in leukemic transformation but rather *SRSF2* is. Progression to BP in all MPN subtypes has been associated with *TP53* mutations and/or loss: such genetic alterations are recorded in up to 35% of patients at transformation, especially in BP occurring in advanced MPN [88,89]. Interestingly, heterozygous *TP53* mutations at a low allelic burden can already be detected during the chronic phase in some patients [12].

### 3.3. Do Less Than 10% Blasts Bear Prognostic Significance?

Interest has recently been devoted to the potential prognostic impact of an increased number of blasts, accounting for less than 10% marrow cellularity in Ph-MPN. Since the progression process is dynamic and gradual, it is conceivable that any increase in the content of immature cells may influence the disease course. In the WHO definition of leukaemic progression [1], blasts accounting less than 10% cellularity is excluded. However, some prognostic schemes, such as IPSS or DIPSS [19,20], do include ≥1% circulating blasts among predictors of disease progression. Indeed, the clinical features of cases with increased blasts falling below the 10% threshold are not well-known. Geyer et al. [92] and Masarova et al. [93,94] recently dealt with this issue, reaching similar conclusions. Cases of PMF showing 5–9% marrow or peripheral blood blasts that were selected among 1.316 patients from the available data, showed comparable OS and clinical parameters, and notably, they resembled those of patients in AP [93]. In addition, these patients showed about a 2-fold rate of leukemic transformation and a lower estimated 3-year LFS rate compared to patients with lower (<5%) blasts. Geyer at al. [92] stratified 92 patients with an initial diagnosis of PMF, PV/post-PV MF, and ET/post-ET MF into three categories according to the blast count: IB-1 (2–4% peripheral blood and <5% bone marrow blasts), IB-2 (5–9% marrow and/or peripheral blood blasts), and AP (≥10–19% marrow or peripheral blood blasts). The IB-2 group had a significantly shorter OS than both the IB-1 group and the controls, while the OS of the combined IB-1/IB-2 groups (*p* = 0.0038) and controls (*p* < 0.0001) was significantly longer than that of patients in the AP group; the IB-1 group OS was similar to that of the controls. These observations are consistent with other publications that have reported that a blast count ≥3% in PMF patients was, among other factors, associated with an increased risk of leukemic transformation in the first 5 years from diagnosis [71,83,95,96]. These data support staining for CD34 in any bone marrow biopsy of chronic phase Ph-MPN. A less than 10% blast increase in bone marrow biopsies that also show cytologic myelodysplasia and some degree of fibrosis in patients with Ph-MPN have occasionally been referred at as myelodysplastic-type progression: these cases morphologically resemble myelodysplasia with fibrosis and have mostly been reported in PV patients [65], some of whom received high-dose P32 and alkylating agents.

## 4. Atypical/Unusual Types of Progression

As referred to above, published data indicate that clinic-pathologic changes can occur during the course of Ph-MPN that cannot be classified according to the present WHO-defined criteria for progression. Still, these conditions may represent examples of bona fide evolution and not simply colliding features, given the reported adverse impact on the clinical course and prognosis. These events shed light on the dynamism and possible overlaps with the different categories of myeloid neoplasms, for which the occurrence of additional transforming events might play a role. The occurrence of monocytosis or leukocytosis (specifically neutrophilia) are rare yet possible events that lead to the acquisition of MDS/MPN–like features (chronic myelomonocytic leukemia/CMML-like and chronic neutrophilic leukemia/CNL-like, respectively): these aspects, in a patient with a previous confirmed history of Ph-MPN, should be described in the pathology report without disease reclassification.

### 4.1. Development of Monocytosis

Monocytosis is reported to develop in 5 to 15% of patients with PMF [85], usually happening years after onset (Figure 1E). If leukoerythroblastosis is present, it commonly persists and shows a higher degree than it does in de novo CMML: this observation may contribute to their clinical differentiation. It is in fact well acknowledged that presence of *JAK2* V617F mutation and monocytosis as well as possible fibrosis can be detected in both CMML and PMF [1]. In these contexts, the patient’s history as well as additional features are crucial to distinguish CMML with a *JAK2* mutation with or without fibrosis from other myeloid neoplasms, particularly PMF. An averagely higher *JAK2* V617F allelic burden (median, 43%; range, 20–62%), the presence of atypical megakaryocytes, and a pronounced myeloid left shift at blood count were reported as being more common in *JAK2* V617F-positive PMF with monocytosis [97]. Multiparametric flow cytometry has been also addressed as a supportive investigation in this differential diagnosis, with a significant difference in the percentage of the so called “MO1 fraction”, namely of the CD14^+^/CD16^−^ monocyte subset. Its value was mostly assessed at ≥94% (mean 95.6%) in CMML cases and at <92% (mean 77%) in MPN patients [98]. Boiocchi et al. [84] reported on 10 PMF patients who had developed persistent monocytosis after a median interval of 42 (range: 1–180) months from diagnosis. Interestingly, in three such cases, the bone marrow biopsy pictures changed after the onset of monocytosis, acquiring a marked myelomonocytic pattern with features that were more consistent with a secondary CMML than a PMF (Figure 1E). The occurrence of monocytosis was reported as an adverse prognostic parameter in patients with PMF or MPN in general by several groups [85,99,100,101,102], and a dose-dependent negative effect on survival has recently been re-confirmed in a large [85] study of 454 PMF patients: this study found that patients who had developed monocytosis were not only more likely to progress to leukemia (HR 2·2, 95% CI 0·99–4·9; *p* = 0·05), and these observations became significant for those with an absolute count >3 × 10^9^/L and who were harbouring *ASXL1* mutations, although the adverse prognostic effect of monocytosis was independent of the mutational status of the driver, the *ASXL1* and *SRSF2* genes; no differences in the distribution of HRM mutations or of an unfavourable karyotype were registered between patients with and without monocytosis.

However, rare cases with bona fide intermediate features between PMF and CMML at onset have been described by Chapman et al. [100]. Once patients with PMF who subsequently developed monocytosis and patients with fibrotic CMML were excluded, six remaining patients were left, most of whom were mostly male and elderly, presenting with monocytosis and organomegaly, PMF-like, and CMML-like hypolobated megakaryocytes in the bone marrow biopsies, with a variable degree of myelodysplasia, marrow fibrosis, and osteosclerosis being present. No myelodysplasia-associated cytogenetic abnormalities were recorded, and all karyotypes were normal. Co-mutations of *JAK2* or *MPL* and *ASXL1*, *SRSF2*, TET2, NRAS, and/or KRAS were detected in five cases where extensive molecular analysis was available. All of the patients progressed. These cases were hardly classifiable according to the WHO criteria: they might represent “atypical” PMF with monocytosis or true “gray zone” cases between Ph-MPN and MDS/MPN. The development of monocytosis has also been described in PV patients by Barraco et al. [101] at a prevalence that was higher than expected. Additionally, in this subset of patients, this event was associated with older age, higher frequencies of leukocytosis, and TET2/*SRSF2* mutations. It also adversely affected OS in univariate analysis and was borderline significant for multivariable analysis as far as OS and myelofibrosis-free-survival were concerned.

### 4.2. Development of Leukocytosis

Leukocytosis is part of the disease history in Ph-negative MPN, and it is less common in ET. A white blood cell increase >15 × 10^9^/L at onset is a risk factor for subsequent thrombosis in both PV and ET patients [103] and a predictor for both inferior survival and leukaemic transformation [104] in PV patients. However, the development of persistent neutrophilia in patients with advanced stage disease (particularly PV) who did not show leukocytosis at onset or during the polycythemic phase has been described as possible MDS/MPN-type progression (namely CNL-like) by Boiocchi et al. [105] in a series of 10 PV patients (Figure 1F). This event was observed shortly before or during fibrotic progression and persisted for a median time of 13 months and invariably ended unfavourably. The neutrophils did not show dysplastic features, although some circulating immature precursors were observed; the bone marrow biopsy pictures, when available, showed a markedly increased myeloid/erythroid ratio with PMF-like or CML-like changes. No modifications of the *JAK2* and BCR-ABL1 status or cytogenetics were recorded. CSF3R, SETBP1, and *SRSF2* were negative.

### 4.3. Development of Erythrocytosis

*JAK2*-mutated ET has rarely been reported to undergo polycythemic transformation, and this has raised the question as to whether this can also be considered a potential type of progression. The reported incidence varies in different studies [6,106]: the lowest value of 1.4% was described by Rotunno et al. [106], and the highest was reported to be 11% by Rumi et al. [6]. This issue was at least partially clarified with the identification of the so-called masked PV [107], which includes patients who are clinically classifiable as ET at onset since they lack PV-specific peripheral blood features but that subsequently increase hemoglobin values during the course of the disease and/or show a more PV- than ET-like bone marrow morphology in the pre-polycythemic phase. The has WHO consistently updated the classification and lowered the blood cut-off value that is necessary for the definition of PV [1]. When these criteria are strictly applied, the incidence of a true polycythemic progression in *JAK2*-mutated ET patients is very low [108], but it is still possible [109]. In these latter cases, no increase in the *JAK2* allele burden was ever observed during the transformation.

## 5. Perspectives and Conclusions

We aimed to provide an updated overview of the types of progression in Ph-chromosome-negative myeloproliferative neoplasms, integrating pathology with the clinical and genetic parameters that impact this step-wise process and of which pathologists should be aware. The molecular data are progressively but rapidly becoming relevant for the management of these patients and are profoundly changing and challenging our diagnostic approach to these diseases. Histology is a turning point in the diagnosis and proper classification of Ph-negative MPN and should also remain as the point of reference in the event of unusual or discordant molecular findings. The immunohistochemical definition of the amount of CD34-positive blasts could be a useful addition in the pathology report of all MPNs in light of the recent literature indicating possible prognosis impact when blasts account for less than 10% marrow cellularity.

The awareness that MPNs are dynamic diseases that may change features over time and that challenge their precise classification is needed. In light of this, it is crucial that all diagnostic aspects that are interpreted in light of the patient’s knowledge and should contribute to the final definition of the disease and its prognosis in a single patient, taking on a fully integrated perspective.

## Figures and Tables

**Table 1:** WHO (revised 4th edition) classification diagnostic criteria for post-ET (**A**) and post-PV MF (**B**).

(A) Diagnostic Criteria for Post-Essential Thrombocythemia Myelofibrosis	
**Required criteria**	Documentation of previous diagnosis of WHO-defined ET
	Bone marrow fibrosis of grade 2–3 on 0–3 scale or 3–4 on a 0–4 scale
Additional criteria (2 are required)	Anemia (i.e., below the reference range given age, sex, and altitude considerations) and a >2 g/dL decrease from baseline hemoglobin concentration
	Leukoerythroblastosis
	Increasing splenomegaly, defined as either an increase in palpable splenomegaly of >5 cm from baseline (distance from the left costal margin) or the development of a newly palpable splenomegaly
	Elevated lactate dehydrogenase level (above the reference range)
	Development of any two (or all three) of the following constitutional symptoms: >10% weight loss in 6 months, night sweats, unexplained fever (>37.5 °C)
**(B) Diagnostic Criteria for Post-Polycythemia Vera Myelofibrosis**	
**Required criteria**	Documentation of previous diagnosis of WHO-defined ET
	Bone marrow fibrosis of grade 2–3 on 0–3 scale or 3–4 on a 0–4 scale
Additional criteria (2 are required)	Anemia (i.e., below the reference range given age, sex, and altitude considerations) or sustained loss of requirement of either phlebotomy (in the absence of cytoreductive therapy) or cytoreductive treatment for erythrocytosis
	Leukoerythroblastosis
	Increasing splenomegaly, defined as either an increase in palpable splenomegaly of >5 cm from baseline (distance from the left costal margin) or the development of a newly palpable splenomegaly
	Development of any two (or all three) of the following constitutional symptoms: >10% weight loss in 6 months, night sweats, unexplained fever (>37.5 °C)
